# Gender differences in coerced patients with schizophrenia

**DOI:** 10.1186/1471-244X-13-257

**Published:** 2013-10-11

**Authors:** Alexander Nawka, Lucie Kalisova, Jiri Raboch, Domenico Giacco, Libor Cihal, Georgi Onchev, Anastasia Karastergiou, Zahava Solomon, Andrea Fiorillo, Valeria Del Vecchio, Algirdas Dembinskas, Andrzej Kiejna, Petr Nawka, Francisco Torres-Gonzales, Stefan Priebe, Lars Kjellin, Thomas W Kallert

**Affiliations:** 1 Department of Psychiatry, First Faculty of Medicine, Charles University, Prague, Czech Republic; 2 Unit for Social and Community Psychiatry, Queen Mary, University of London, London, UK; 3 Department of Psychiatry, University of Naples, Naples, Italy; 4 Central land office, Ministry of Agriculture, Prague, Czech Republic; 5 Department of Psychiatry, Medical University of Sofia, Sofia, Bulgaria; 6 Psychiatric Hospital, Thessaloniki, Greece; 7 School of Social Work and Geha Mental Health Center, University of Tel Aviv, Tel Aviv, Israel; 8 Psychiatric Clinic, Vilnius Mental Health Centre, University of Vilnius, Vilnius, Lithuania; 9 Department of Psychiatry, Medical University, Wroclaw, Poland; 10 Psychiatric private practice, Dresden, Germany; 11 Department of Legal Medicine and Psychiatry, Medical Faculty, University of Granada, Granada, Spain; 12 Psychiatric Research Centre, Orebro County Council, Orebro, Sweden; 13 School of Health and Medical Sciences, Orebro University, Orebro, Sweden; 14 Department of Psychiatry, Psychosomatic Medicine, and Psychotherapy, Park Hospital Leipzig, Leipzig, Germany; 15 Soteria Hospital Leipzig, Leipzig, Germany; 16 Department of Psychiatry and Psychotherapy, Faculty of Medicine, Dresden University of Technology, Dresden, Germany

**Keywords:** Gender, Schizophrenia, Involuntary treatment, Seclusion, Restraint, Forced medication, Aggressive behavior, EUNOMIA project

## Abstract

**Background:**

Despite the recent increase of research interest in involuntary treatment and the use of coercive measures, gender differences among coerced schizophrenia patients still remain understudied. It is well recognized that there are gender differences both in biological correlates and clinical presentations in schizophrenia, which is one of the most common diagnoses among patients who are treated against their will. The extent to which these differences may result in a difference in the use of coercive measures for men and women during the acute phase of the disease has not been studied.

**Methods:**

291 male and 231 female coerced patients with schizophrenia were included in this study, which utilized data gathered by the EUNOMIA project (European Evaluation of Coercion in Psychiatry and Harmonization of Best Clinical Practice) and was carried out as a multi-centre prospective cohort study at 13 centers in 12 European countries. Sociodemographic and clinical characteristics, social functioning and aggressive behavior in patients who received any form of coercive measure (seclusion and/or forced medication and/or physical restraint) during their hospital stay were assessed.

**Results:**

When compared to the non-coerced inpatient population, there was no difference in sociodemographic or clinical characteristics across either gender. However coerced female patients did show a worse social functioning than their coerced male counterparts, a finding which contrasts with the non-coerced inpatient population. Moreover, patterns of aggressive behavior were different between men and women, such that women exhibited aggressive behavior more frequently, but men committed severe aggressive acts more frequently. Staff used forced medication in women more frequently and physical restraint and seclusion more frequently with men.

**Conclusions:**

Results of this study point towards a higher threshold of aggressive behavior the treatment of women with coercive measures. This may be because less serious aggressive actions trigger the application of coercive measures in men. Moreover coerced women showed diminished social functioning, and more importantly more severe symptoms from the “excitement/hostile” cluster in contrast to coerced men. National and international recommendation on coercive treatment practices should include appropriate consideration of the evidence of gender differences in clinical presentation and aggressive behaviors found in inpatient populations.

## Background

Since the advent of psychiatry as a profession, coercive measures have been used in the treatment of mental illness. Although there has been a recent increase of interest in involuntary treatment [1-5], gender differences among coerced patiets still remain understudied [6,7].

It is well recognized that gender differences have an impact on mental health in many disorders, and in particular on the course of schizophrenia [8]. Neurodevelopmental [9,10] and neuropathological theories [11,12], and the estrogen protection hypothesis [13,14] have all been proposed as explanations for how gender differences develop in schizophrenia. Taken together, these three theoretical frameworks can integrate a wide variety of findings of gender differences in schizophrenia, with compelling evidence existing for all approaches [15,16].

The way that gender modifies the phenotypic expression of schizophrenia is a consistent theme in the literature, and a number of gender differences have been reported [17]. Schizophrenia onset occurs at a significantly earlier age in male patients in comparison to female patients [15,18,19]. Male patients are more severely impaired in ratings of negative symptoms [18,20,21], cognitive impairment [15], express less severe positive symptoms [18] and are more likely to show severe deterioration over time [19]. Female patients are likely to have more severe positive symptoms [19], with more hallucinations [22], persecutory delusions [15], affective symptoms [15,19] and greater number of suicide attempts [22]. Women also show lower pre-morbid cognitive performance [23] and the course of the illness is considerably less severe [24]. In contrast to research summarized above, several studies have shown no gender difference in symptom severity [25], neurocognitive functioning [25,26], delusional symptoms [22], positive symptoms [20], minor physical anomalies [15] or neurological soft signs [15].

The course of the illness is more favorable in females in the short- [27] and middle-term [15], with females manifesting better social functioning [20,24,28] and having fewer hospitalizations with shorter inpatient stays [24,29]. Women with schizophrenia are also more often married [25,30], employed [25] and live independently [25]. Males have poorer premorbid functioning [15,22,31], are frequently unemployed, live alone [19,22] have poorer social networks [22] and poorer functional outcome [18]. Only one study, conducted in Australia, found no gender difference in social functioning [31]. The duration of untreated psychosis has been found to be similar for both genders [22].

Psychotic disorders, including schizophrenia, are the most common diagnosis among patients who are involuntarily admitted to psychiatric hospitals and treated against their will [32]. Aggressive behavior and poor insight play a major role in involuntary (re)hospitalization [33]. Ries et al. [34] found that 65% of a population of acutely admitted patients with schizophrenia were males. Man with schizophrenia have been found to commit severe acts of violence more frequently than woman [35,36]. However, less severe aggressive behavior, for example verbal threats, has been found to be more frequent among women [37,38]. Other studies have found no gender differences in aggressive behavior among patients with schizophrenia [39,40].

It is possible that gender differences in the clinical presentation, and biological correlates of severe mental illness may result in a different use of coercive measures during the acute phases of psychiatric disorders and in hospitalizations for men and women [41,42]. Studies have found that physical restraint was used more often with male patients, while forced medication and seclusion was used more often with female patients [41]. Male gender is also associated with higher rates of seclusion [43], restraint [44] and psychiatric intensive care [45]. Other studies have found that physical restraint was more often used with females [42] and female patients were more frequently secluded than their male counterparts [46]. No gender differences in the use of mechanical restraint have been found in studies conducted in the USA [47], or Finland, where all the forms of coercive measures studied were equally commonly applied to male and female patients [48,49]. Approval rates of coercive methods however, are greater by male than by female patients [45,50]. This finding was also replicated in the EUNOMIA study, where females expressed more negative views on whether involuntary admission was right or wrong [5].

The aim of this study was to investigate whether there are gender differences among coerced patients with schizophrenia in 12 European countries, in regards to the sociodemographical and clinical characteristics, social functioning, aggressive behavior, and the use of coercive measures.

## Methods

This study utilized data gathered by the EUNOMIA project (European Evaluation of Coercion in Psychiatry and Harmonization of Best Clinical Practice). The main objective of the EUNOMIA project was to analyze the existing national variations in coercive psychiatric treatment within the European region, along with its influencing factors and outcomes. The characteristics of the individual centers, including the number of hospital beds per 100 000 inhabitants, the number of staff per bed and the average number of beds per room were assessed by the European Service Mapping Schedule, vision 3 [51] and a special instrument describing the characteristics of hospitals [52] which were published in our previous article [7]. A more detailed description of the EUNOMIA project methodology might be found elsewhere [1-3,5,7]. The EUNOMIA project was carried out as a multi-centre prospective cohort study at 13 centers in 12 European countries: Dresden, Germany; Sofia, Bulgaria; Prague, Czech Republic; Thessaloniki, Greece; Tel Aviv, Israel; Naples, Italy; Vilnius, Lithuania; Wroclaw, Poland; Michalovce, Slovak Republic; Granada and Malaga, Spain; Orebro, Sweden; and East London, United Kingdom; and each participating center recruited involuntarily admitted patients between July 2003 and December 2005. Patients who fulfilled the following criteria were included in the analysis: diagnosis of schizophrenia (i.e., F20.0-F20.9 diagnosis according to ICD-10 as established by psychiatric reports within the first seven days of admission); patient has received any form of coercive measure (seclusion and/or forced medication and/or physical restraint) during their hospital stay, age between 18 and 65 years; ability to sign an informed (written) consent form; not admitted to a special unit for only forensic or intoxicated patients; not included in the study before (repeated admissions during the study period); not transferred to a participating clinic from another hospital; and having a permanent living address in the catchment area of the participating hospitals.

Patients who fulfilled the criteria were assessed by researchers at three different points in time: within the first seven days of admission (T1), at 4 weeks or at discharge (T2), and at 3 months after admission (T3), independent of the patient’s current living situation. Baseline socio-demographic characteristics and diagnosis of the patient according to ICD-10 [53] (WHO 1998) were obtained from medical records. These included data on age, gender, civil status, living situation, employment situation, previous psychiatric hospitalizations and previous involuntary hospitalizations. As an indicator of clinical functioning, symptom levels were assessed on the 24-item version of the Brief Psychiatric Rating Scale (BPRS) [54], which ranges from 24 to 168, with 168 indicating the maximum symptom severity. Global Assessment of Functioning scale (GAF) [55] was used as an indicator of global social functioning. This scale constitutes axis V of the Diagnostic and Statistical Manual for Mental Disorders 4th edition (DSM-IV) [56] and assesses patient’s social occupational and psychological functioning in a hypothetical continuum of 1 to 100 points, which is divided in 10 ranges of 10 points, although a single score that represents patient’s level of functioning is obtained. All researchers were trained to use both scales. Inter-rater reliability for BPRS scale was assessed throughout the project (videotaped interview on the international level and with personal interviews on the national level) and an inter-rater reliability with interclass correlation coefficient of 0.78 was achieved. As for the training on GAF scale, 72 GAF vignettes were jointly rated by researchers that had received first a local training session and then a common international video training session (in English). The GAF inter-rater reliability for the whole training process was good with an interclass correlation coefficient of 0.74. The Modified Overt Aggression Scale (MOAS), a widely used aggression scale with documented reliability and validity, was used to evaluate violent behavior for the duration of hospitalization. The scale has four categories of aggressive behavior (verbal aggression, aggression against property, auto-aggression, and physical aggression) [57]. Data concerning details of each application of coercive measures during the first 4 weeks of hospitalization or up to his/her discharge were gathered using a special 16-item questionnaire designed by the EUNOMIA group for the purpose of this study [7]. Coercive measures were defined as follows: 1) seclusion: the involuntary placement of an individual locked in a room alone, which may be set up especially for this purpose; 2) restraint: fixing at least one of the patient’s limbs with a mechanical device or being held by a staff member for longer than 15 minutes; and 3) forced medication: activities using restraint or strong psychological pressure (involving at least three staff members) to administer medication against the patient’s will. Informed consent was obtained from all patients in this study after they were provided a complete description of the study. The national or regional review boards of the participating centers approved the study:

Research Ethics Committee, Medical University Sofia, Sofia, Bulgaria

The Ethics Committee of the General Teaching Hospital, Prague, Czech Republic

Ethics committee at the Faculty of Medicine at Dresden University of Technology, Dresden, Germany

Scientific Board of the Psychiatric Hospital of Thessaloniki, Thessaloniki, Greece

The Tel Aviv University IRB-Helsinki Committee, Tel Aviv, Israel

Ethical Committee of the Second University of Naples, Naples, Italy

Lithuanian Bioethics Committee, Vilnius, Lithuania

Commission of Bioethics at Wroclaw Medical University, Wroclaw, Poland

Ethical Committee of the Michalovce Psychiatric Hospital, Michalovce, Slovak Republic

Ethical Committee (Comité Ético) of University Hospital of San Cecilio. Granada, Spain

Research Ethics Committee of Örebro University Hospital, Örebro, Sweden

East London and The City Research Ethics Committee, London, UK

All statistical analyses were performed using SPSS version 17.0. *T*-test were performed to identify differences in socio-demographic factors between male and female patients. Differences between the groups in categorized background variables were assessed by chi-square analysis or, where appropriate, Fisher’s exact tests. Correlation analysis and binary logistic regression were used for evaluation of how clinical characteristics, social characteristics and aggressive behavior were associated with gender. Logistic regression was used to estimate bivariate and adjusted association ratios for the dichotomous sex outcome categories. The candidate explanatory variables for a multiple regression were screened with uni-variate ordinal logistic regression. A main effect multivariable model was applied. Chi square test, Mann Whitney test, *t*-test were used to assess bivariate associations.

## Results

Initially 1284 involuntary patients with schizophrenia were identified and of those final sample of coerced patients recruited in this study included 291 male and 231 female patients (total=522; 55.8% vs. 44.2%). 74.6% males and 64.0% females were patients with paranoid type of schizophrenia (F20.0). Patients with residual type of schizophrenia (F20.5) were the second largest group with 21.2% being women and 12.0% men. Undifferentiated type of schizophrenia (F20.3) accounted for 12.1% female and 8.6% male patients. Other types of schizophrenia: disorganized (F20.1); catatonic (F20.2); and other or unspecified schizophrenia types (F20.8 and F20.9) together represented only 4.8% of male, and 2.7% of female patients.

Female patients were significantly older (41.1 ± 10.8) than their male counterparts (35.7 ± 10.8) (p < .05). Men were significantly more likely to be single (77,0% vs. 41.2%) while women were more likely to be married (30.3% vs. 14.4%), divorced (22.2% vs. 8.6%) or widowed (6.3% vs. 0%) (p < .001). Female patients lived on their own significantly more often than male patients (70.5% vs 46.1%). Almost half of male patients (48.4%) reported living with their family/partner/friend, compared to only 26.7% of women (p < .001). Only 1.4% male and female patients lived in social institutions and the proportion of homeless was also very low (2.4% males and 0.9% females). Male patients were significantly more likely to be unemployed (41.0% vs. 29.2%), but the numbers of those partially or fully employed (20.0% vs. 19.6%) did not differ among genders. The biggest proportion of both genders, however, was on social welfare (33.1% males and 43.0% females).

There was no significant difference in respect to the past hospitalizations. About one-quarter of male patients and one-fifth of female patients had been admitted for the first time, and over three-quarters from both genders had been re-hospitalized. No significant difference among genders was found in respect to the past involuntary hospitalizations (*Χ*^2^ = 0.12, df = 1, p = .73), but due to partial data availability no reliable proportions could be calculated. The BPRS total score, as an indicator of overall severity of symptoms, was significantly higher for female patients (58.9 ± 14.5 vs. 54.6 ± 14.0) (p = .004) at T1. When performing an in depth analysis of individual items of BPRS several gender differences were apparent. Female patients scored significantly higher on several items, as follows. From the “positive cluster” [58]: hallucinations (3.15 ± 2.0 vs. 2.80 ± 1.8) (p < .001); bizarre behavior (3.28 ± 1.7 vs. 2.80 ± 1.7) (p < .001); conceptual disorganization (2.57 ± 1.6 vs. 2.27 ± 1.5) (p < .001). From the “negative cluster”: emotional withdrawal (2.37 ± 1.4 vs. 2.10 ± 1.3) (p < .001). Finally, from the “activation/manic cluster”: uncooperativeness (2.29 ± 1.6 vs. 2.03 ± 1.5) (p < .001); and motor hyperactivity (2.51 ± 1.7 vs. 1.94 ± 1.3) (p < .001). Male patients did not score significantly higher on any of the individual items. A similar pattern of results to those found for BPRS were observed when comparing GAF scores as measures of global social functioning, suggesting more severe impairment in women. Male patients scores were significantly higher (30.5 ± 12.7 vs. 26.2 ± 12.8) (p < .001), indicating better social performance (Table 1). Table 2 shows bivariate and adjusted association for the main effect model of independent variables on gender categories. Clinical characteristics among coerced patients, according to BPRS subscales discriminate to a certain level between genders. More severe psychopathology in the “positive psychotic” subscale is associated with the male category, and “active/manic” and “negative psychotic” subscales with the female category. Overall global functioning also discriminated between sex categories, showing higher scores for men category.

**Table 1:** Socio-demographic, clinical and social functioning characteristics of the sample at the baseline

	**Total sample**	**Female**	**Male**	***p***
	**N = 522 (%)**	**N = 231 (%)**	**N = 291 (%)**	
Age (mean±SD)	38.1 ± 11.1	41.1 ± 10.8	35.7 ± 10.8	*t-test, p < .001*
Type of schizophrenia				
Paranoid schizophrenia	365 (70.0)	148 (64.0)	217 (74.6)	*Χ*^*2*^* = 14.441, df = 6, p = .025*
Psychiatric hospitalization in the past	389 (74.5)	178 (79.5)	211 (74.0)	*Χ*^*2*^* = 2.052, df = 1, p = .152*
Marital status				
Single	319 (61.1)	95 (41.2)	224 (77.0)	*Χ*^*2*^* = 75.514, df = 3, p < .001*
Employment status				
Unemployed	186 (35.6)	67 (29.2)	119 (41.0)	*Χ*^*2*^* = 24.969, df = 6, p < .001*
Housing situation				
Live on their own	311 (59.6)	205 (70.5)	106 (46.1)	*Χ*^*2*^* = 46.516, df = 5, p < .001*
BPRS total score (mean±SD)	56.5 ± 14.7	58.9 ± 14.5	54.6 ± 14.0	*t-test, p = .004*
GAF score (mean±SD)	28.6 ± 12.9	26.2 ± 12.8	30.5 ± 12.7	*t-test, p < .001*

**Table 2:** Logistic regression analysis of effects on gender categories

**Independent variables**	**Bivariate associations**		**Main effects model**	
	***OR***	***95% CI***	***OR***	***95% CI***
Positive psychotic	0.96*	0.93-0.99	1.06*	1.01-1.12
Suspiciousness/hostility	0.95*	0.91-0.99	NS	
Active/manic	0.92*	0.89-0.96	0.95*	0.9-0.99
Depression/anxiety	NS		NS	
Negative psychotic	0.94*	0.91-0.97	0.95*	0.9-0.99
Baseline GAF	1.02*	1.01-1.04	1.03*	1.01-1.05

*p < .005.

More than two-thirds of both groups, men and women, have developed aggressive behavior during the first four weeks after admission (79.6% females and 71.7% males). When assessing aggressive behavior simply by counting average MOAS scores for both groups, no significant difference was found (females 5.20 ± 5.61 vs. males 5.62 ± 6.80) (p = .462). Women were more likely to show aggressive behaviors but with a lesser intensity (total MOAS score 1 to 7) (50.2% vs. 40.2%) and men were found to be more severely aggressive when counting only those who scored 8 or higher in MOAS (14.47 ± 5.61 vs. 12.34 ± 4.97) (p = .01) (Table 3). 373 incidents were recorded of coercive measures being applied to 231 women and 573 to 291 men during the first four weeks of the hospitalization. The most frequently used coercive measure was forced medication (80.7%), followed by physical restraint (57.1%) and seclusion (10.7%). Women were more likely to receive forced medication (87.9% vs. 74.9%) (odds ratio (OR) 2.4, 95% confidence interval 1.51-3.90), whereas men were more likely to be physically restrained (66.2% vs. 45.5%) (OR = 2.4, CI 1.66-3.67) or secluded (17.2% vs. 2.6%) (OR = 7.8, CI 3.27-18.50) (p < .001). No significant difference has been observed in the reasons that led to the use of coercive measures. From those provided in this study the most common reasons in both genders were “to prevent acts of violence against others” (56.0% females and 59.0% males), followed by “worsening of condition” (31.4% females and 27.8% males), and by “aggression against objects” (23.6% vs. 18.5%) (Table 4).

**Table 3:** Aggressive behavior observed during the hospital stay

		**Female**	**Male**	***p***
		**N = 231 (%)**	**N = 291 (%)**	
Verbal aggression	Total	169 (73.2)	192 (67.1)	*Χ*^*2*^* = 2.203, df = 1, p = .138*
	Severe^¥^	18 (7.8)	60 (21.0)	*Χ*^*2*^* = 17.346, df = 1, p < .001*
	Average score	1.57 ± 0.69	1.88 ± 1.00	*p = .015*^*#*^
Aggression against property	Total	79 (34.2)	72 (25.5)	*Χ*^*2*^* = 4.593, df = 1, p = .032*
	Severe^¥^	7 (3.0)	26 (9.2)	*Χ*^*2*^* = 8.082, df = 1, p = .004*
	Average score	1.5 ± 0.77	2.0 ± 0.96	*p < .001*^*#*^
Auto-aggression	Total	25 (10.8)	34 (11.8)	*Χ*^*2*^* = .123, df = 1, p = .726*
	Severe^¥^	5 (2.2)	12 (4.2)	*Χ*^*2*^* = 1.622, df = 1, p = .203*
	Average score	1.76 ± 0.97	2.32 ± 1.01	*p = .023*^*#*^
Physical aggression	Total	95 (41.3)	115 (39.8)	*Χ*^*2*^* = .122, df = 1, p = .727*
	Severe^¥^	10 (4.4)	21 (7.3)	*Χ*^*2*^* = 1.943, df = 1, p = .163*
	Average score	1.47 ± 0.74	1.66 ± 0.84	*p = .09*^*#*^
MOAS total score 1–7 (mean±SD)		116 (50.2)	117 (40.2)	*p = .58*^*#*^
		3.18 ± 1.92	3.13 ± 2.17	
MOAS total score 8 or higher (mean±SD)		67 (29.0) 12.34 ± 4.97	83 (28.5) 14.47 ± 5.61	*p = .01*^*#*^
MOAS total score (mean±SD)		5.20 ± 5.61	5.62 ± 6.80	*p = .462*^*#*^

^¥^scores 3 or 4 on respective MOAS items.

^#^Mann–Whitney test.

**Table 4:** Coercive measures used in involuntary treated psychotic patients and the reasons for their use

		**Female**	**Male**	**p**
		**N = 231 (%)**	**N = 291 (%)**	
Type of coercive measure used	Forced medication	203 (87.9)	218 (74.9)	*Χ*^*2*^* = 13.871, df = 1, p < .001*
	Physical restraint	105 (45.5)	193 (66.3)	*Χ*^*2*^* = 22.892, df = 1, p < .001*
	Seclusion	6 (2.6)	50 (17.2)	*Χ*^*2*^* = 28.602, df = 1, p < .001*
Total number of coercive measures applied		N = 373	N = 573	*Χ*^*2*^* = 0.07, df = 1, p = .78*
Reasons for the use of coercive measures (chosen)	Prevent acts of violence against her/himself	74 (19.8)	95 (16.6)	*Χ*^*2*^* = 1.636, df = 1, p = .224*
	Severe danger or threat for his or her health	117 (31.4)	159 (27.8)	*Χ*^*2*^* = 1.432, df = 1, p = .242*
	Inability to care for him-/herself	34 (9.1)	62 (10.8)	*Χ*^*2*^* = .720, df = 1, p = .441*
	Prevent acts of violence against others	209 (56.0)	338 (59.0)	*Χ*^*2*^* = .809, df = 1, p = .382*
	Prevent acts of violence against property	88 (23.6)	106 (18.5)	*Χ*^*2*^* = 3.595, df = 1, p = .059*
	Prevent escape	49 (13.1)	88 (15.4)	*Χ*^*2*^* = .900, df = 1, p = .347*

## Discussion

This is the first international multicenter study which assessed gender differences across a large sample of coerced, involuntary treated patients with schizophrenia using standardized instruments. A number of pertinent findings can be drawn from our results: 1) across neither gender do coerced patients differ in sociodemographic or clinical characteristics from the non-coerced inpatient population; 2) coerced female patients show a worse social functioning than their male counterparts, which is in contrast to the non-coerced inpatient population; 3) patterns of aggressive behavior are different between men and women; women exhibit aggressive behavior more frequently, but men commit severe aggressive acts more frequently. These gender differences in behavior may lead, along with “cultural factors” to 4) different patterns in the use of coercive measures among genders, where forced medication is the preferred method for women, while physical restraint and seclusion is used more frequently for men.

Males accounted for 55.8% of the patients and females for 44.2%, which closely replicates the findings from a study of 1755 involuntary admitted patients in the USA (57.8 vs. 42.2) [32] and a study of 2222 patients in Denmark (63.6% vs. 36.4%) [59]. In Salize´s report the percentage of compulsory admitted male patients varied between 50% in Sweden and 69% in France [60].

The study sample consisted mainly patients with paranoid schizophrenia (with slight prevalence of men) and residual schizophrenia (with slight prevalence of women). The finding that majority of people within the residual subtype of schizophrenia were women is in contrast with studies reporting a larger number of men evolving into residual schizophrenia, most likely because of greater frequency of negative symptoms and multiple admissions [61]. It is possible that the age difference between genders in our sample of involuntary admitted patients accounts for this difference, with women being older than men. Collectively, the other types of schizophrenia accounted for less than fifteen percent of our sample across both genders.

Consistent with the findings of other studies about the sociodemographic characteristics of “voluntary” treated patients with schizophrenia [22,25], our study found no major differences between voluntarily and involuntarily treated patients on sociodemographic variables. Coerced women were significantly older than men; they were more likely to be married, divorced or widowed; lived on their own more frequently; and were less frequently unemployed. Coerced men were significantly more likely to be single; lived more frequently with their families, partners or friends; and were more frequently unemployed. Half of all patients of both genders were on social welfare. The proportion of the homeless psychotic patients who have been treated involuntarily was very low across both genders, which contrasts with findings from a US study, which found the proportion of homeless people in their sample to be ten times higher [62]. The chronicity of the psychotic illness was clearly associated with the likelihood of receiving coercive measures. More than three-quarters of all patients in our sample were re-hospitalized and the vast majority of women and men had already experienced involuntary hospitalization. Overall, female patients showed more severely impaired clinical functioning in comparison to men. Women scored higher than men on measures of several individual positive symptoms such as hallucinations and bizarre behavior, which is in line with other studies on schizophrenia populations [15,19,22]. However male gender was associated with overall higher scores on the “positive psychotic” subscale. Furthermore, coerced women were not more severely delusional than men and also did not score more highly on measures of affective symptoms, findings that converge with those described in other gender studies with non-coerced schizophrenia patients [15,22]. Coerced women did, however, score more highly than coerced men on the “negative symptom” emotional withdrawal, which differs to findings described elsewhere in non-coerced populations [18,20,21]. Women also scored more highly on two symptoms from the “excitement/hostile cluster”; uncooperativeness and motor hyperactivity, a fact that is mirrored in their more frequent involvement in aggressive behavior.

Our findings show differences in theclinical manifestations between the coerced population studied in this sample and other non-coerced schizophrenia populations. Further, and of greater interest, is the finding that coerced females showed a significantly worse social functioning than coerced men. This finding is of particular interest, as the majority of studies dealing with schizophrenia populations have reported the opposite finding, women showing higher social functioning than men [20,24].

Aggressive behavior is very common among involuntarily admitted patients with schizophrenia. In this study female patients were involved in almost 80% in some kind of aggressive behavior, while men in slightly more than 70%. Although this difference wasn´t significant this finding may suggest a discrepancy with the literature on violence in out-patient psychiatric populations, where men are more violent than women. There are several possible factors that may explain this discrepancy. Firstly, assaults in men are associated with substance abuse, property crime and school truancy [63], factors that have little influence in the inpatients setting. Secondly, the presence of major mental disorders, including schizophrenia, increases the risk for violent offending relatively more in women than in men [64]. These results converge with those of studies showing that male overrepresentation vanishes in inpatient psychiatric populations [65]. In one study hospitalized women patients committed more assaults than their male counterparts, although men engaged in more fear-inducing behavior [66]. These results are consistent with our findings, where female patients were more frequently aggressive but with lesser intensity, whereas males were responsible for the most severe aggressive acts. These results have been observed for overall aggression as well as for verbal aggression and aggression against property items in MOAS. Interestingly, if only average scores for the aggression as measured by the MOAS instrument were used, no significant difference among genders would have been detected.

Although some studies have found no association between the risk of being coerced and gender [48,49] in psychiatric populations, our study revealed several differences in the use of coercive measures. In European institutions men with schizophrenia were more than twice likely to end up being physically restrained than women, while the opposite pattern was true for the use of forced medication. One can only speculate on the reasons for such difference. One possible explanation for the higher use of forced medication among women may be the higher recorded rates of positive psychotic symptoms, especially considering positive psychotic symptoms are more likely to result in assaults in women than in men [63]. The most credible explanation for the more frequent use of physical restraint in men is that staff may feel more threatened by more serious aggressive behavior by men in comparison to the same aggressive behavior by women. Physical restraint may be seen as a more immediate way to control this aggression, and thus be considered a “safer” option to avoid aggressive acts against the hospital staff and other patients. However, as Lam et al. [65] conclude, there is an equal likelihood of injuries to staff members being caused by violent female patients as by violent male patients and thus signs of an elevated risk of violence should not be ignored on the basis of gender. The use of seclusion differed strongly across genders, with the likelihood of men being secluded being almost eight times higher than for women. It is possible that this large difference in the use of seclusion may again be explained by the more severe aggressive behavior observed in males (although it should be noted that seclusion was not used in all centers). Clearly cultural and local traditions and legislative practices will play an crucial role in how specific coercive measures are applied [1,2]. For example in the Netherlands involuntary medicating is highly restricted, while mechanical restraint is being forbidden in the UK [67]. When questioned about coercive measures for a Norwegian study, younger male patients reported, that they would prefer physical restraint and older male patients reported preferring seclusion over forced medication, whereas forced medication was preferred by female patients [41]. These findings were confirmed by a study conducted in the Netherlands, where female patients reported preferring forced medication over seclusion, while males preferred seclusion over forced medication [68]. In England physical restraint was strongly disapproved of by both genders [45], and female patients were more frequently subject to seclusion than their male counterparts and they were secluded more often but for shorter periods [46]. In a study by Georgieva et al., women reported that they had experienced coercive interventions as more burdensome than men [69], which may reflect their greater emotional responsiveness and lower average tolerance thresholds for painful stimuli [70]. In the future, instruments which measure the psychological impact of psychiatric coercive interventions, such as the “Coercion Experience Scale” [71] should be used to how compare different coercive interventions are experienced by patients. No significant difference was observed in the reasons given for the use of coercive measures. The most common reason reported for both genders was “to prevent acts of violence against others”, followed by “worsening of condition”, and by “aggression against objects”. Auto-aggressive behavior accounted for less than one-fifth of reasons given for the use of coercive measures, and surprisingly no gender differences were found either, which contrasts with other research, reporting women with schizophrenia as making a greater number of suicide attempts [22].

When interpreting the findings of this study some limitations must be taken into account. The recruitment process in this type of research is widely recognized as being very difficult (involving recruitment of severely ill patients with schizophrenia who are involuntary admitted and experiencing coercive measures) and therefore although only half of the eligible patients were interviewed, at rate that has been described as good for this type of study (acute settings with difficult-to-recruit patients) [72]. There is only limited data available to compare recruited and non-recruited patients for the United Kingdom, but analysis of the available data does not suggest a selection bias on the assessed characteristics [5]. Data on the use of coercive measures were based on available documentation and additional sources from the site personnel. The documentation of coercive measures in clinical records may differ between participating countries, and the number of unrecorded or unreported measures may also vary. However, all centers used a uniform and standardized protocol for data collection and the process of gathering all available information was rigorously applied. Although it was usual for several inpatient units in each country to be involved in this study [7], the data can´t be fully generalizable because the variability in the use of coercive measures between hospitals in the same country is high [73].

## Conclusion

A number of our findings have implications for the education of hospital staff, including the finding that the overall frequency of aggressive behaviors by schizophrenia patients is actually higher in women than it is in men, but the most severe aggressive behaviors are more frequent in men. Results of this study also point towards a higher threshold at which women are treated with the use of coercive measures. It is possible that this may be due to less serious aggressive actions by men triggering the application of coercive measures, as aggressive behavior by men is perceived as more threatening than the same behavior expressed by women. Moreover coerced women, in comparison with their non-coerced female counterparts and in contrast to coerced men, have diminished social functioning, and more importantly more severe symptoms from the “excitement/hostile” cluster.

Delineating gender differences in the use of coercive measures in patients with schizophrenia is important for developing targeted treatments [22,74]. Therefore national and international recommendation on coercive treatment practices should include appropriate consideration of the evidence of gender differences in clinical presentation and aggressive behaviors found in inpatient populations.

## Competing interests

The authors declare that they have no competing interest.

## Authors’ contributions

JR, GO, AKa, ZS, AF, VDV, AD, AKi, PN FT, SP, LK and TWK participated in the design of the study. AN, LK, DG were involved in the data collection. AN, LK, DG and LC helped draft the manuscript and partially participated in the design of the study. LC performed the statistical analysis. All authors read and approved the final manuscript.

## Pre-publication history

The pre-publication history for this paper can be accessed here:

http://www.biomedcentral.com/1471-244X/13/257/prepub
